# Supplementary figures and images for: Nitrogen and Silicon Contribute to Wheat Defense’s to Pyrenophora tritici-repentis, but in an Independent Manner
Source: Plants (Basel). 2024 May 21;13(11):1426. doi: 10.3390/plants13111426 (PMC11174962; doi:10.3390/plants13111426)

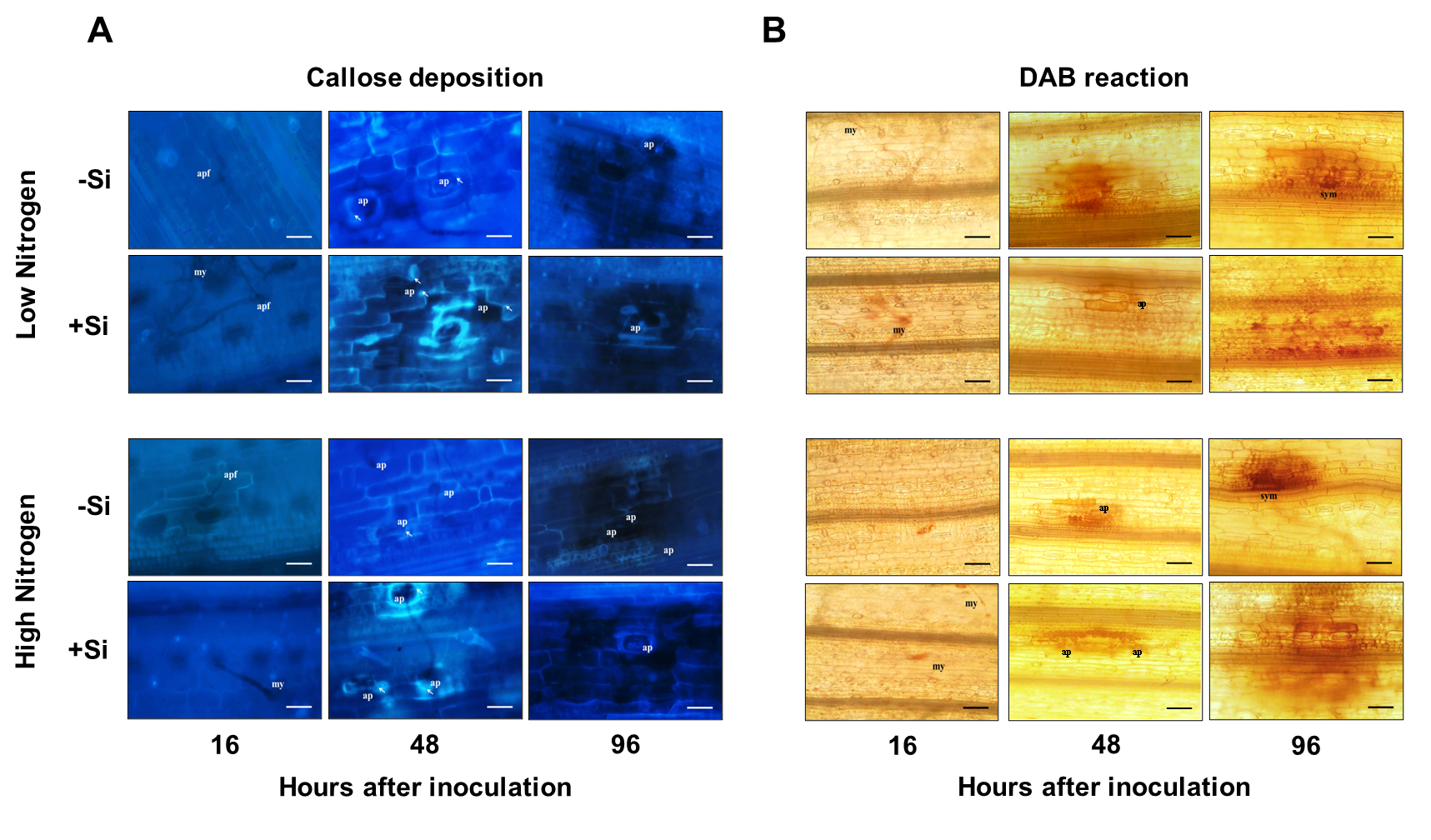

Supplement: Supplementary file 1 [file plants-13-01426-s001.zip › plants-2995836-supplementary.png]
